# Prenatal diagnosis of genetic aberrations in fetuses with pulmonary stenosis in southern China: a retrospective analysis

**DOI:** 10.1186/s12920-023-01548-1

**Published:** 2023-05-30

**Authors:** Meiying Cai, Nan Guo, Meimei Fu, Yuqing Chen, Bin Liang, Yanting Que, Qingqiang Ji, Hailong Huang, Liangpu Xu, Na Lin

**Affiliations:** Medical Genetic Diagnosis and Therapy Center, Fujian Maternity and Child Health Hospital College of Clinical Medicine for Obstetrics & Gynecology and Pediatrics, Fujian Medical University, Fujian Key Laboratory for Prenatal Diagnosis and Birth Defect, Fuzhou, China

**Keywords:** Pulmonary stenosis, Pathogenic copy number variation, Chromosomal microarray analysis, Pregnancy outcome

## Abstract

**Background:**

The genetic etiology of congenital pulmonary stenosis (PS) in fetuses remains inadequately studied. We used karyotype analysis and chromosomal microarray analysis (CMA) to investigate the genetic aberrations associated with PS in human fetuses.

**Methods:**

A retrospective analysis was performed on 84 fetuses with congenital PS in southern China. Fetal amniotic fluid and umbilical cord blood samples were obtained for chromosomal karyotype analysis and CMA.

**Results:**

The rate of pathogenic copy number variation (CNV) was 15.5% (13/84) after karyotyping and CMA. An abnormal karyotype was detected in five cases (6.0%, 5/84) via karyotyping, whereas pathogenic CNVs were detected in 13 cases (15.5%, 13/84) via CMA. In addition to the five abnormal karyotypes detected using karyotype analysis, eight additional chromosomal microduplications and microdeletions were detected using CMA, comprising three cases of 22q11.21 microdeletion; two cases of 16p11.2 microdeletion; one case of simultaneous 18q23 microdeletion and 22q13.33 microduplication; one case of 15q24.1q24.2 microdeletion; and one case of 1q21.1q21.2 microduplication. The rate of pathogenic CNV occurrence was 11.5% in fetuses with isolated PS and 17.2% in fetuses with PS combined with other ultrasound abnormalities. This difference between the two experimental groups was statistically significant. Among 84 fetuses with PS, 39 pregnancies were terminated, and five were lost to follow-up.

**Conclusions:**

CMA was not only conducive to detect PS-related pathogenic genomic abnormalities but also to accurately evaluate fetal prognosis in genetic counseling. The early detection of PS and genomic abnormalities will exerta positive impact on fetal intervention and the related prognosis of PS in perinatal infants.

## Background

Pulmonary stenosis (PS) comprises a combination of abnormal pulmonary valve development, subvalvular stenosis, and (rarely) supra pulmonary stenosis with stenosis of the pulmonary trunk and its branches, resulting in the obstruction of right ventricular cardiac outflow [1, 2]. PS is fairly common, accounting for 10–20% of all congenital heart diseases, and can occur either in isolation or alongside other cardiac and external malformations [3]. Despite the ubiquity of PS, few studies have investigated its etiology in fetuses for the evaluation of postnatal outcomes. An effective prenatal examination of PS pathophysiology would enable doctors to evaluate the fetal status in a timely and comprehensive manner to prepare for subsequent treatment.

Chromosomal microarray analysis (CMA), which detects chromosomal microdeletion and microduplication, has been applied to the prenatal diagnosis of individuals with developmental delays, intellectual disabilities, and a variety of congenital abnormalities [4–10]. This technique can detect copy number variations (CNVs) at > 100 kb, rendering it superior to traditional cytogenetic karyotyping (which is limited to detection at > 10 Mb) [11, 12]. This study aimed to screen the whole genome DNA of fetuses with PS for candidate pathogenic genes related to PS based on their CNVs. The prenatal diagnosis of PS is of considerable importance in guiding clinical practice, improving prognosis, and optimizing prenatal and postnatal care related to this common disease.

## Methods

### Patient data

Over 30, 000 fetuses received prenatal diagnosis from November 2016 to January 2022 in southern China. Our inclusion criterion was confirmation of a fetal PS phenotype, either with or without additional structural abnormalities, via prenatal ultrasound for karyotyping and CMA simultaneously. The exclusion criterion was the lack of a PS phenotype, or the fetuse with PS receiving prenatal diagnosis only for karyotyping. Ultrasonography detected 84 fetuses with PS, and amniotic fluid or umbilical cord blood was extracted from the pregnant women carrying these fetuses to conduct prenatal diagnoses. The mean age of these women was 29 ± 1 years, and the mean gestational age was 24 ± 5 weeks. The 84 fetuses were divided into an isolated PS group (n = 26) and a non-isolated PS group (n = 58), based on the presence or absence of other ultrasound abnormalities. This study was approved by the Ethics Committee of Fujian Maternal and Child Health Hospital in China, and informed consent was obtained from all parents of the fetuses selected for inclusion in the study.

### Karyotype analysis

The amniotic fluid or cord blood samples of 84 fetuses were cultured, harvested, prepared, and G-banded (C- and N-banding were added, if necessary) according to conventional methods. Karyotype collection and karyotype analysis were performed using a GSL-120 automatic chromosome-scanning platform. Forty karyotypes were counted for each sample, among which five karyotypes were analyzed. The karyotype count and subsequent number of analyses were increased in cases of abnormality.

### CMA

Our experiment was conducted in strict accordance with all standard procedures provided by Affymetrix for sample genomic DNA extraction, digestion, amplification, purification, fragmentation, labeling, hybridization with chips, washing, scanning, and data analysis. A CytoScan HD microarray (Affymetrix) was used, with the chip containing both single-nucleotide polymorphism and oligonucleotide probes. Corresponding Chromosome Analysis Suite software and bioinformatics methods were used to analyze the CMA detection results. Chromosome microdeletion and microduplication were determined according to the scatter plot distribution of DNA copy number. For our comparative analysis of CNVs, we included both internal and online public databases based on the existing literature and the clinical significance of CNVs > 100 kb in public databases. These sources comprised the Database of Genomic Variance(http://projects.tcag.ca/variation/), DECIPHER (http://www.sanger.ac.uk/PostGenomics/decipher), Online Mendelian Inheritance in Man (http://www.omim.org), and the University of California Santa Cruz database (http://www.genome.UCSC.edu/). CNVs can becategorized into pathogenic, likely pathogenic, likely benign, and benign CNVs and variants of uncertain clinical significance (VUSs), depending on their nature [13].

### Follow-up

The outcomes of all pregnancies related to this study were followed up telephonically. The health of all infants was also followed up until they reached 1 year of age. Postpartum diagnoses and prognoses were recorded.

### Statistical analysis

SPSS 20.0 software was used for all statistical analyses. Comparisons between groups were performed using thechi-square test or Fisher’s exact probability method. Statistical significance was set at P < 0.05.

## Results

### Chromosome karyotyping

Abnormal karyotypes were detected in five of the 84 (6.0%) fetuses with PS. They comprised four aneuploid abnormalities (three cases of trisomy 21 and one case of 47, XXY) and one unbalanced translocation of large fragment chromosomes (Table 1). The parents of the fetuses bearing these abnormal karyotypes received genetic counseling and chose to terminate the respective pregnancies.

**Table 1:** Results ofkaryotype analyses of fetuses with PS

Case	Karyotype	CMA	Prenatal ultrasound	Postnatal outcome
1	47,XN,+21	arr[hg19] (21)x3	PS, FGR, Endocardial pad defect, Right aortic arch, Persistent left superior vena cava, Nasal bone dysplasia	TP
2	47,XN,+21	arr[hg19] (21)x3	PS, VSD, Aortic straddle	TP
3	47,XN,+21	arr[hg19] (21)x3	PS, VSD, Aortic straddle, Nasal bone dysplasia	TP
4	47,XXY	arr[hg19] (X)x2,(Y)x1	PS, VSD,Polycystic renal dysplasia, Nasal bone dysplasia	TP
5	45,XN,-18,+mar	arr[hg19]18p11.32p11.31(136,227-3,348,254)x1,18p11.31p11.21(3,350,736 − 13,083,388)x3,18p11.21(13,090,666 − 15,170,636)x1,18p11.21q21.31(15,181,20754,008,143)x3,18q21.31q23(54,020,488 − 78,013,728)x1	PS, VSD,Aortic coarctation	TP

FGR,fetal growth restriction; VSD, ventricular septal defect; TP, termination of pregnancy; PS, pulmonary stenosis

### CMA

Among the 84 samples, 17 displayed abnormal CMA results, comprising 13 cases of pathogenic CNVs (15.5%) and four cases of VUSs (4.8%, 4/84) (Table 2). The 13 pathogenic CNV cases consisted of four instances of aneuploidy, one of unbalanced translocation of large fragment chromosomes, and eight of chromosome microduplication or microdeletion. The eight cases displaying chromosomal microduplications or microdeletions comprised three instances of the microdeletion of 22q11.21, two of the microdeletion of 16p11.2, one of simultaneous 18q23 and 22q13.33 microdeletion, one of 15q24.1q24.2 microdeletion, and one of 1q21.1q21.2 microdeletion. Following genetic counseling, the parents of the eight fetuses with PS carrying these eight pathogenic CNVs opted to terminate the respective pregnancies.

**Table 2:** CMA results of fetuses with PS

Case	CMA	Size (Mb)	Prenatal ultrasound	Pathogenicity classification	Postnatal outcome	Inheritance
1	arr[hg19]22q11.21(18,631,364 − 21,800,471)x1	3.1	PS, VSD, Aortic straddle	P	TP	*denovo*
2	arr[hg19]22q11.21(18,648,855 − 21,800,471)x1	3.1	PS, VSD, Aortic straddle	P	TP	*-*
3	arr[hg19]22q11.21(18,919,477 − 21,800,471)x1	2.8	PS	P	TP	*denovo*
4	arr[hg19]16p11.2(29,428,531 − 30,177,916)x1	0.7	PS	P	TP	*denovo*
5	arr[hg19]16p11.2(29,428,531 − 30,190,029)x1	0.6	PS, VSD, Aortic straddle	P	TP	*denovo*
6	arr[hg19]1q21.1q21.2(145,995,176 − 147,398,268)x3	1.4	PS,Tricuspid stenosis, Right heart dysplasia	P	TP	*maternal*
7	arr[hg19]18q23(73,969,018–78,013,728)x1, 22q13.33(49,571,996 − 51,197,766)x3	4.0, 1.6	PS	P	TP	
8	arr[hg19]15q24.1q24.2(72,965,465 − 75,567,135)x1	2.6	PS, VSD, FGR, Nasal bone dysplasia	P	TP	*denovo*
9	arr[hg19]15q13.3(32,003,537 − 32,444,043)x3	0.4	PS, VSD, Aortic straddle Right aortic arch with mirroring branch	VUS	TP	*-*
10	arr[GRCh37]3p26.2p25.1(2886527_13828221)x2hmz,4p16.3p15.33(3473602_14373371)x2hmz,5p13.3p11(31554333_46313469)x2 hmz	10.9, 10.9, 14.8	PS,Strephenopodia	VUS	TD	*-*
11	arr[hg19]8p22(17,179,780 − 18,518,478)x3	1.3	PS, VSD, Aortic straddle	VUS	TP	*-*
12	arr[hg19]16p12.3(19,333,917 − 20,487,932)x1	1.1	PS, VSD, Aortic straddle,Single umbilical artery	VUS	TP	*-*

FGR,fetal growth restriction; VSD, ventricular septal defect; P, pathogenic; TP, termination of pregnancy; TD, term delivery; PS, pulmonary stenosis; CMA, chromosomal microarray analysis

### Comparison of karyotyping and CMA results

The combined detection rate of karyotyping and CMA was 15.5% (13/84). In addition to the five abnormalities detected via karyotyping, nine additional chromosomal microduplications and microdeletions were detected via CMA. The detection rates of pathogenic CNVs in the two groups were 6.0% (5/84) and 15.5% (13/84), respectively, and there was a statistically significant difference between the two groups (X^2^ = 3.982, P = 0.046, P < 0.05).

### Comparison of pathogenic CNV detection between the two experimental groups

Among the 26 cases inthe isolated PS group, we detected three instances of pathogenic CNVs (a positivity rate of 11.5%). In comparison, among the 58 cases in the non-isolated PS group, 10 instances of pathogenic CNVs were detected (a positivity rate of 17.2%). Although the positivity rate of pathogenic CNVs was higher in the non-isolated than in the isolated PS group, this difference was not statistically significant (X^2^ = 0.117, P = 0.732, P > 0.05).

### Pregnancy outcome and follow-up

Our 84 cases of PS were followed up to establish pregnancy outcomes and record postnatal development. We successfully followed up on 79 cases; five cases were lost to follow-up. Among the 79 cases that were followed up successfully, 39 fetuses were induced and consisted of 13 with pathogenic CNVs, three with VUSs, and 23 with normal CNVs. The postnatal growth and development of newborns from the remaining 40 cases exhibiting a normal CNV and one child carrying VUSs were assessed to be normal. Among them, 29 cases were followed up only telephonically. However, 11 newborns underwent echocardiographic examination, and the results of these tests were consistent with those obtained via our prior ultrasonic phenotyping of the respective fetuses. Three of these infants received cardiac surgery and were subsequently followed up for approximately another 6 months, during which all were assessed to be in a good condition (Table 3).

**Table 3:** Pregnancy outcomes of 84 fetuses with PS

CMA	TP (n)	Pregnancy TD (n)	Outcomes TD,echocardiography (n)	TD,cardiac surgery (n)	Loss follow-up (n)
pCNV	13	0	0	0	0
VUS	3	1	0	0	0
Normal	23	28	8	3	5

CMA, chromosomal microarrayanalysis; pCNV, pathogeniccopy number variation; VUSs, variants of uncertain clinical significance; TP, termination of pregnancy; PS, pulmonary stenosis

## Discussion

Many disease-causing genes associated with birth defects have been identified using CMA [14–16]. In prenatal diagnoses, CMA can detect chromosomal microdeletions and/or microduplications and identify associated pathogenic genes in fetuses, with ultrasonography being able to reveal concurrent structural abnormalities. To explore the relationship between CNV and PS in human fetuses, this study applied CMA to fetuses with a confirmed prenatal diagnosis of PS. The CNVs of these fetuses with PS were screened for disease-causing genes that may be related to PS. Of the 84 fetuses with PS screened via CMA, 15.5% (13/84) possessed pathogenic CNVs.

Among the 13 cases of pathogenic CNVs detected by CMA, eight possessed microdeletion/microduplication syndrome, of which 22q11.2 and 16p11.2 microdeletions were more common. At present, there are many reports on the microdeletion of 22q11.2 and 16p11.2 being related to congenital heart disease; however, these diseases primarily constitute ventricular septal defects, and reports of PS are rare [17–20]. The findings of this study suggest that the 22q11.2 microdeletion and 16p11.2 microdeletion are related to PS. In addition, four other rare syndromes were detected in this study: lq21.1, 18q23, 22q13.33, and 15q24. The lq21.1 microduplication syndrome represents a rare CNV with major clinical manifestations that include intellectual disability, autism, pediatric acromegaly, and congenital heart disease. The lq21.1 microdeletion is more closely associated with congenital heart disease than the lq21.1 microduplication [21]. However, in recent years, several researchers have begun to focus on the correlation between lq21.1 microduplication and congenital heart disease. Soemedi et al. [22] studied the chromosome rearrangement of the lq21.1 segment in 2,436 patients with congenital heart disease and found that lq21.1 duplication is more common in tetralogy of Fallot, whereas lq21.1 microdeletion is more common in other types of congenital heart disease. Prenatal ultrasounds in our study revealed PS, tricuspid stenosis, and right heart dysplasia in fetuses. CMA showed 1.4 Mb of microduplicates in the 1q21.1–q21.2 region, which contains the *GJA5* gene, potentially linking the pathology of fetal PS and other congenital heart defects with this gene. In support of this hypothesis, Gu et al. [23] demonstrated that mice with a *GJA5* allele deletion exhibited a series of congenital heart defects, including PS. Patients with 15q24 microdeletion syndrome are primarily characterized by intrauterine growth delay, short stature, intellectual disability, microcephaly, and congenital heart defects, among other symptoms [24]. In this study, the prenatal ultrasound phenotypes of one fetus with 15q24 microdeletion syndrome were consistent with such symptoms, consisting not only of PS but also a ventricular septal defect, fetal growth restriction, and nasal bone dysplasia. Both 18q23 microdeletion and 22q13.33 microduplication syndromes present with a variety of clinical manifestations including heart or gastrointestinal malformations, microcephaly, growth restriction, and abnormal facial features [25, 26]. However, PS was the only prenatal ultrasound phenotype of one fetus in this study with both 18q23 microdeletion and 22q13.33 microduplication syndrome.

In this study, a fetus possessed a VUS CNV that CMA identified to be a 15q13.3 microduplication. Ultrasound imagery of this fetus revealed PS, a ventricular septal defect, an aortic straddle,and a right aortic arch with a mirroring branch. The clinical phenotype of the 15q13.3microduplication may include language impairment, cognitive impairment, developmental delay, epilepsy, schizophrenia, special facial features, and other abnormalities [27–29]. However, congenital heart defects were not reported in the afore mentioned literature. The *CHRNA7* gene is contained within the 15q13.3 region, and an increased presence of this gene correlates with individual cognitive and behavioral phenotypes [30]. Whether the *CHRNA7* gene dose is related to heart malformations requires further investigation.

Our CMA results showed that the rate of pathogenic CNVs was 11.5% in fetuses from the isolated PS group and 17.2% in fetuses from the non-isolated PS group; however, this difference was not statistically significant. Therefore, karyotype analysis and CMA can be recommended for fetuses with isolated ornon-isolated PS. However, the rate of pathogenic CNV detection differed significantly between karyotype analysis (6.0%) and CMA comparative analysis (15.5%) of the 84 samples in this study. Therefore, the detection of the genetic etiology via CMA is strongly recommended when the ultrasound phenotype of a fetus is PS.

Previous studies have indicated that fetuses with mild and moderate PS have a good prognosis after birth [31, 32]. In the follow-up of patients with mild PS, they exhibited little change in the pulmonary valve pressure; in some children, the pulmonary valve pressure difference even decreased or returned to normal with an increase in age. In this study, the growth and developmentof newborns with PS and a normal CNV were determined to be normal during postnatal follow-up assessments.When known chromosomal microdeletions or microduplications were excluded, parents were more confident in opting to continue their pregnancies. Three babies were diagnosed with severe PS via postnatal echocardiography and underwent surgical treatment at 1 month. After 6 months of further follow-up, they displayed good postoperative recovery. Therefore, pregnancies should not automatically be terminated when fetuses are diagnosed with PS and a normal CNV. Echocardiographic evaluation should be conducted as soon as possible after birth in these cases, allowing for timely surgery.

This study has certain limitations, including the lack of whole-exon sequencing in fetuses with PS and those with normal CNVs [33–35]. Whole-exon sequencing can detect more single-gene mutations and thus improve the detection rate of genetic causative factors, which is more comprehensive than CMA. In addition, among the over 30, 000 fetuses studied for our prenatal diagnosis, only 84 displayed congenital pulmonary artery stenosis; the small sample size may have affected the study’s findings. Therefore, additional investigations employing a larger study population is critical to clarify the genetic pathogenesis and etiology of PS.

## Conclusions

CMA was applied as a prenatal diagnostic tool for PS in human fetuses. The technique was conducive to the detection of PS-related pathogenic genomic abnormalities, providing an accurate fetal prognosis that facilitated the genetic counseling of parents. Early detection of PS and timely detection of genomic abnormalities will exerta positive impact on the intervention of fetuses with PS and prognosis of perinatal infants. The etiology of PS can be effectively prevented in advance, and the incidence of PS can be reduced in the near future.

## Data Availability

The microarray data from this study were submitted to the Gene Expression Omnibus repository (https://www.ncbi.nlm.nih.gov/geo/query/acc.cgi?acc=GSE208291).

## Abbreviations

CMA: Chromosomal microarray analysis
CNV: Copy number variation
PS: Pulmonary stenosis
VUSs: Variants of uncertain clinical significance
